# Rich resource environment of fish farms facilitates phenotypic variation and virulence in an opportunistic fish pathogen

**DOI:** 10.1111/eva.13355

**Published:** 2022-02-25

**Authors:** Katja Pulkkinen, Tarmo Ketola, Jouni Laakso, Johanna Mappes, Lotta‐Riina Sundberg

**Affiliations:** ^1^ 4168 Department of Biological and Environmental Science University of Jyväskylä Jyväskylä Finland; ^2^ Research Programme in Organismal and Evolutionary Biology, Faculty of Biological and Environmental Sciences, University of Helsinki Helsinki Finland; ^3^ 4168 Nanoscience Center University of Jyväskylä Jyväskylä Finland

**Keywords:** aquaculture, bacterium, colony type, fish diseases, phenotypic variation

## Abstract

Phenotypic variation is suggested to facilitate the persistence of environmentally growing pathogens under environmental change. Here, we hypothesized that the intensive farming environment induces higher phenotypic variation in microbial pathogens than natural environment, because of high stochasticity for growth and stronger survival selection compared to the natural environment. We tested the hypothesis with an opportunistic fish pathogen *Flavobacterium columnare* isolated either from fish farms or from natural waters. We measured growth parameters of two morphotypes from all isolates in different resource concentrations and two temperatures relevant for the occurrence of disease epidemics at farms and tested their virulence using a zebrafish (*Danio rerio*) infection model. According to our hypothesis, isolates originating from the fish farms had higher phenotypic variation in growth between the morphotypes than the isolates from natural waters. The difference was more pronounced in higher resource concentrations and the higher temperature, suggesting that phenotypic variation is driven by the exploitation of increased outside‐host resources at farms. Phenotypic variation of virulence was not observed based on isolate origin but only based on morphotype. However, when in contact with the larger fish, the less virulent morphotype of some of the isolates also had high virulence. As the less virulent morphotype also had higher growth rate in outside‐host resources, the results suggest that both morphotypes can contribute to *F. columnare* epidemics at fish farms, especially with current prospects of warming temperatures. Our results suggest that higher phenotypic variation per se does not lead to higher virulence, but that environmental conditions at fish farms could select isolates with high phenotypic variation in bacterial population and hence affect evolution in *F. columnare* at fish farms. Our results highlight the multifaceted effects of human‐induced environmental alterations in shaping epidemiology and evolution in microbial pathogens.

## 1 INTRODUCTION

Anthropogenic modifications in the environment, such as changes in temperature, precipitation, or nutrients, affect species in many ways and cause adaptations suited to the prevailing regime of changes (Kristensen et al., 2020). For pathogens, changes in host availability and survival of environmental stages of pathogens have the potential to drive evolution of pathogen traits via anthropogenic change (Mideo & Reece, 2012; Wolinska & King, 2009). One example of human‐induced change in the selective environment for pathogens is intensive farming (Mennerat et al., 2010; Pulkkinen et al., 2010). The epidemiological conditions at farms that select for fast growth and high transmission between hosts in pathogens are likely to select for different genotypic and/or phenotypic properties than the conditions outside the farming environment (Mennerat et al., 2010; Wolinska & King, 2009). For example, intensive farming has been associated with increases in virulence (harm to the host) for pathogens such as Marek´s disease virus in poultry farming (Atkins et al., 2013) as well as salmon lice (Mennerat et al., 2010, 2012; Ugelvik et al., 2017) and *Flavobacterium columnare* in aquaculture (Pulkkinen et al., 2010; Sundberg et al., 2016).

Phenotypic plasticity is one of the mechanisms that allow organisms to adjust to changing environments (Ackermann, 2015; Nussey et al., 2007; Rainey & Travisano, 1998; Scheiner, 1993; Via et al., 1995). The ability of a single genotype to produce different phenotypes increases the genotype's fitness under environmental change and ensures persistence in fluctuating environments (Ackermann, 2015; Via et al., 1995). Energetic costs associated with maintaining the metabolic machinery or trade‐offs between different phenotypic properties in different environments may limit the adaptive value of phenotypic variation (DeWitt et al., 1998; Koch & Guillaume, 2020). However, these trade‐offs may be relaxed if a resource‐rich environment enables stronger allocation to several costly functions simultaneously (van Noordwijk & de Jong, 1986). The costs of phenotypic responses depend also strongly on ambient temperature due to temperature dependence of metabolic rates (Huey & Kingsolver, 2011). In addition, predictability and the speed of the environmental change determine the profitability of phenotypic change (DeWitt et al., 1998; Kristensen et al., 2008; Kussell & Leibler, 2005; Padilla & Adolph, 1996). A randomly changing environment increases the cost of plasticity due to a mismatch between the signal for phenotypic change and the realized environmental conditions (Reed et al., 2010; Tonsor et al., 2013). In such unpredictable environments, risk‐spreading strategies, like bet‐hedging, are expected to evolve (Botero et al., 2015; DeWitt & Langerhans, 2004; Levins, 1968). In this paper, we use the term phenotypic variation to describe a genotype's ability to change phenotype, irrespective of whether it is due to an environmental cue, or due to random switching of phenotypes (bet‐hedging).

Phenotypic variation might be especially important for opportunistic pathogens that survive and replicate not only within the host but also in the outside‐host environment (Brown et al., 2012; Ketola et al., 2016). The available ecological opportunities outside the host can differ vastly from those encountered by the pathogen inside the host (Anttila et al., 2016; Brown et al., 2012). Within the host, the greatest challenges are posed by the host immune system (Schmid‐Hempel, 2009). In the outside‐host environment, low availability of resources, presence of predators, parasites and competitors, and abiotic factors, such as temperature, often restrict the growth (Adiba et al., 2010; Friman et al., 2009, 2011; Hibbing et al., 2010; Ketola et al., 2013; Zhang et al., 2014). The ability to change from one alternative phenotype to another with different characteristics, for example, for competitive ability or immune evasion might be crucial for the expression of pathogenicity for opportunistic pathogens (Holland et al., 2014; Ketola et al., 2016; Kreibich & Hardt, 2015).

In bacteria, phenotypic variation is often visible in the form of different types of colony morphologies, with differences in growth characteristics in the bacterial population forming the colony (Friman et al., 2009; Koh et al., 2007; Kunttu, Suomalainen, et al., 2009; Rainey & Travisano, 1998). Phenotypic variation in bacteria may rise as a response to change in environmental conditions or as a consequence of stochastic switching between alternative phenotypes produced by the same genotype (Ackermann, 2015).

Intensive farming represents an environment that has been heavily modified by human actions. For pathogens, intensive farming represents an environment with ample possibilities but also challenges for growth and survival. On one hand, the high density of genetically homogeneous hosts and excess feed offers abundant resources for growth, but on the other hand, medical treatments during outbreak season pose a recurrent and strong threat for pathogen survival (Atkins et al., 2013; Mennerat et al., 2010; Pulkkinen et al., 2010). For most of the time in the natural environment, the microbes persist outside of the hosts with few opportunities for fast growth. At farms, high host availability may favor phenotypes capable of fast exploitation of hosts, that is, high virulence (Ebert, 1998; Frank, 1996). On the other hand, periodical medical treatments targeted to diseased hosts may favor phenotypes that can escape treatments by growing on organic material in the outside‐host environment. The ability of environmentally growing pathogens to persist and replicate outside the host is expected to lead to increased virulence as pathogen reproductive success is not restricted by host death; that is, there is no trade‐off between virulence and transmission (Day, 2002; Ewald, 1994).

Our study focused on an opportunistic fish pathogen *Flavobacterium columnare*, a cause of severe economic losses to fish production worldwide (Declercq et al., 2013; Wagner et al., 2002). The virulence of the pathogen has been suggested to have increased during the last few decades at fish farms in Finland (Ashrafi et al., 2018; Pulkkinen et al., 2010; Sundberg et al., 2016). At Finnish fish farms, the disease outbreaks occur only during summer, when water temperatures reach *c*.*a*. 18°C (Pulkkinen et al., 2010). Disease outbreaks start when waterborne bacterial cells proliferate in alive or dead fish tissue, or other organic material in the water (Kunttu et al., 2012; Kunttu, Valtonen, et al., 2009). Increased resource levels in the outside‐host environment increase virulence in the bacterium via increased doses and virulence factor activation (Kinnula et al., 2017; Penttinen et al., 2016) and could increase the contribution of less virulent strains in outbreaks (Pulkkinen et al., 2018).

*Flavobacterium columnare* changes colony morphotype from a spreading rhizoid (Rz) type to a nonspreading rough (R) type either spontaneously or after exposure to virulent phages, with a concomitant decrease in virulence (Laanto et al., 2012; Sundberg et al., 2014). The two colony types also differ in growth parameters and responses to protozoan predators, with suggested costs of expressing R type in the outside‐host environment (Zhang et al., 2014). The spontaneous change of colony types expressed by *F. columnare* is reversible (Laanto et al., 2012). While the exact mechanisms leading to colony morphology changes are not known, and the abundance and the possible role of the R type in the environment are unclear, the different colony types are suggested to serve different functions in invasion and replication in fish and the outside‐host environment (Kunttu, Suomalainen, et al., 2009; Kunttu, Valtonen, et al., 2009; Laanto et al., 2014; Zhang et al., 2014).

We hypothesized that large changes in environments associated with intensive farming facilitate higher phenotypic variation in environmentally growing microbial pathogens than the more stable natural environment. Increased resource availability in intensive farming environments promotes large population sizes and could also decrease the costs associated with phenotypic variation (DeWitt et al., 1998; Friman et al., 2008) and increase the probability of phenotypic variation. We hypothesized that *F. columnare* isolates originating from fish farms would present higher phenotypic variation in growth and virulence than isolates from natural waters. We also hypothesized that due to resource‐ and temperature‐driven growth, these differences would be more pronounced in higher resources and higher temperatures. To test the hypothesis, we measured the growth of two morphotypes (ancestral rhizoid, Rz, and its rough, R, derivative, Figure S1) of each of five isolates originating from both environments. We took measurements in five different resource concentrations and two temperatures, relevant for occurrence of disease epidemics at farms (Ashrafi et al., 2018; Pulkkinen et al., 2010). In addition to maximal growth rate, we also analyzed bacterial yield as a potentially relevant trait for bacterial persistence at farms. Due to potential costs in expressing the different morphotypes (Zhang et al., 2014), we also tested the stability of the morphotypes in plate cultivation under the same resource concentrations that were used for the growth measurements. Finally, we tested phenotypic differences between the natural and the fish farm isolates in bacterial virulence in fish challenge tests in vivo.

## 2 MATERIALS AND METHODS

### 2.1 *Flavobacterium* *columnare* colony morphologies

*Flavobacterium columnare* exhibits three different colony morphologies, of which the rhizoid type (Rz) is associated with high virulence (Kunttu, Suomalainen, et al., 2009). The other two types, rough (R) and soft (S), are associated with lower virulence (Kunttu, Suomalainen, et al., 2009; Laanto et al., 2012). Primary isolations from natural samples on selective agar plates (Decostere et al., 1997) produce generally a rhizoid colony morphology, while the two other types appear during continued re‐cultivation (Kunttu, Suomalainen, et al., 2009; Sundberg et al., 2014), or as a response to infection by phages (Laanto et al., 2012). In this study, we compared the ancestral Rz type and its R derivative formed spontaneously in subcultivation.

### 2.2 Isolation and cultivation of bacteria

Fish farm isolates were obtained from diseased fish or tank water during outbreaks at fish farms as part of disease surveillance. The isolates from nature were collected from water or a diseased wild fish upstream of a fish farm and they represent the variation present in natural waters (Ashrafi et al., 2015; Kunttu et al., 2012) (Table 1). Bacteria isolations were performed with standard culture methods, using modified Shieh medium (from now on: Shieh medium) (Song et al., 1988) supplemented with tobramycin (Decostere et al., 1997). Pure cultures were stored at −80°C with 10% glycerol and 10% fetal calf serum.

**TABLE 1:** The *Flavobacterium columnare* isolates used in this study

Isolate identity	Genotype	Location	Month/year isolated	Source
B392	ND^a^	Off‐farm V^e^	06/2010	Fish *Abramis brama*, natural waters
B394	A	Off‐farm V^e^	06/2010	Fish *Abramis brama*, natural waters
B399	G/ST7^b^	Off‐farm V^e^	06/2010	Natural waters
B408	C	Off‐farm V^e^	08/2010	Natural waters
Tulo2	A	Off‐farm V^e^	08/2010	Natural waters
B402	C	Farm V^e^	2010	Fish (*Coregonus lavaretus*)
B425	NA^c^	Farm V^e^	2007	Fish (*Oncorhynchus mykiss*)
B067	A	Farm L^e^	2007	Fish (*Salmo trutta*)
Jip39/87 (ATCC 49513)	GenI^d^	Type strain, France	1987	Fish (*Ictalurus melas*)
E	E	Farm O^e^	2002	Fish (*Salmo salar*)

Note

Genotyping of Finnish strains is based on ARISA (Suomalainen et al., 2006), or MLSA analysis, which associates uniformly with ARISA typing (Ashrafi et al., 2015).

^a^ ND not determined, falls outside previously determined genetic groups of Finnish strains, see (Ashrafi et al., 2015), additional file; isolate B392 has only allele 4.

^b^ See Ashrafi et al. (2015), additional files 1 and 6.

^c^ NA not analysed.

^d^ See Ashrafi et al. (2015), Fig. 2.

^e^ See Laanto et al. (2012).

All isolates exhibited originally a rhizoid morphotype. Rough colonies that appeared spontaneously during plate cultivation were collected singly and cultured in Shieh medium at room temperature with constant shaking (210 rpm) and plated to assure the loss of parental Rz growth before storing at −80°C (Figure S1).

Before the experiments, bacteria were revived from frozen stocks by inoculation to Shieh medium (1× concentration). Bacteria were cultured at room temperature with constant agitation (210 rpm) for 24 h and subsequently enriched in 1/10 dilution in fresh Shieh medium overnight in the same conditions. For growth measurements at different resource concentrations, 10 µl of the enriched culture was applied into 100‐well Bioscreen C^®^ plates containing 400 µl of fresh Shieh medium at different concentrations (2×, 1×, 0.5×, 0.1×, and 0.05× of Shieh medium). Maximum growth rates and yield were determined from biomass growth data recorded at 5 min intervals with 420–580 nm optical density for 6 days at 25°C and 10 days at 15°C. Each isolate–morphotype combination was included twice into separate measurements on two subsequent weeks, totaling four replicate measurements. In addition, after liquid culture, bacterial samples were plate cultured on Shieh agar with the five different resource concentrations mentioned above, to check the morphotype stability under different resource levels.

### 2.3 Virulence experiments

The fish experiments were conducted according to the Finnish Act of the Use of Animals for Experimental Purposes, under permission ESAVI‐2010‐05569/Ym‐23 granted by the National Animal Experiment Board at the Regional State Administrative Agency for Southern Finland for L‐RS.

For testing virulence, we used zebrafish (*Danio rerio*), which have been established as a reliable model system for revealing differences in virulence among strains in *F. columnare* (Kinnula et al., 2015). Zebrafish were obtained from Core Facilities (COFA) and research services of Tampere University, Finland. The fish used in the experiment were disease‐free, adult, and unsexed. The weight range of fish challenged with fish farm isolates and natural isolates were 0.06–0.88 g and 0.08–0.87 g, respectively. For each isolate–morphotype combination, 10 individual fish were challenged, with 10 additional fish used as controls, totaling 210 individual fish. The fish were infected using continuous immersion challenge (Kinnula et al., 2015). Fish were placed individually in 1‐L plastic containers filled with 500 ml of aerated well water at 25°C with freshly grown bacteria added in 4 ml of fresh Shieh medium to reach a final bacterial concentration of 1 × 10^5^ CFU ml^−1^ in the container. For control fish, 4 ml of Shieh was added without bacteria. Fish were monitored for 4 days for disease symptoms and morbidity. Morbid fish that did not respond to external stimuli were considered dead, removed, put down by cutting the cordial spine with scissors, and weighed. At the end of the experiment, all remaining fish were terminally euthanized with MS‐222. The presence of *F. columnare* infection on fish was checked by plating a primary culture from the gills onto modified Shieh agar plates.

### 2.4 Data analysis

The maximum growth rates of the bacteria were assessed from the OD data. For calculating the growth parameters, we used all the data points concerning 25°C data as there were no apparent lag phases, whereas with the 15°C data the estimation of the maximum growth rate was started after 2 h to exclude the lag phase, that is, no visible growth after inoculation. First, we log‐transformed OD data, which linearizes the exponential growth. This allows finding the point of fastest growth rate using linear regression, by sequential fitting of 25 (or other desired) data point sliding windows to the data. From all subsets of 25 data points, we sought the subset with the steepest regression slope that equals the maximum growth rate. Maximum biomass, that is, yield was determined as the largest found averaged OD value over the subset. The MATLAB code to perform these analyses is described in Ketola et al. (2013). The point estimates of maximum growth rate and yield were further analyzed with linear models.

As the dataset on bacterial growth contained several factors that could interact in many ways, we utilized model selection to reduce the complexity of the models explaining growth rate or yield. We started model building from the model containing all possible interactions between measurement temperature (T), resource concentrations (R), origin of the isolate (O), and morphotype (M) (all fixed factors). In all models, we included “maternal” clone identity as a random factor to control for nonindependence resulting from four measurement replicates, and from the fact that colony types originated from the same bacterial culture. Moreover, the effect of measurement day was fitted as a fixed factor in all models. Starting from the highest order interactions, each of the interactions was removed from the model, if the interaction was not statistically significant (based on *p*‐value > 0.10) and its removal improved the model fit (AIC). The same selection procedure was used to test how experimental factors explain biomass yield. Since yield is often found to trade‐off with growth rate (Velicer & Lenski, 1999), we tested also if our results concerning growth rate could be explained by the inclusion of yield as a covariate. To explore whether yield has similar effects in all treatment groups, we fitted also interactions of the covariate (standardized to mean of zero) with all factors (Hendrix et al., 1982). The same model selection as above was utilized to drop out nonsignificant factors to covariate interactions. Since covariate and factor by covariate interactions did not affect the significance of our results or conclusions, it is clear that changes between treatments are not caused by life history trade‐offs between maximal growth rate and yield. Thus, these results are not presented or considered further. Statistical testing was performed with REML mixed models with IBM SPSS v. 19 (IBM).

In fish challenge experiments, the fish were monitored for 96 h and the last moribund fish was encountered and euthanized at 54 h. Thus, the remaining challenged fish were considered as true survivors, and not as censored cases. Therefore, we used generalized linear mixed models for binomial distribution to examine the morbidity caused by different morphotypes of replicate strains originating from nature or fish farms. We analyzed the morbidity risk of a host in an hour with a model including all possible interactions between the origin of the isolate (O; nature, fish farm), colony type (T; rhizoid, rough), and fish weight (W; as a continuous covariate). Strain identity (S) was included as a random factor. Model reduction based on AIC criteria was performed with drop1‐function in package MASS (Venables & Ripley, 2002) starting from the full model. The analysis was conducted using R software (version 3.3.2) and the Lme4 package (R Development Core Team, 2015). To be able to visualize data fitting accuracy in Figure 5, we rerun the best model using equivalent model but using Bayesian methods (Stan Development Team, 2020). Posterior values of estimates were used to calculate estimates of mortality risks given the fish weight and the colony morphology. To formally test the region of fish weight where rhizoid and rough colony types differ in their mortality risk, we utilized a method analogous to Johnson‐Neyman procedure (Hayes & Matthes, 2009), where the posterior distribution of mortality risks at each fish size (i.e., predicted values for 0.01‐g intervals), for rhizoid and rough, were compared for posterior distribution overlap. This way we approximated the critical weight, below which the strain morphology causes differences in mortality (*p* < 0.05).

## 3 RESULTS

### 3.1 Growth rate

**FIGURE 1:**
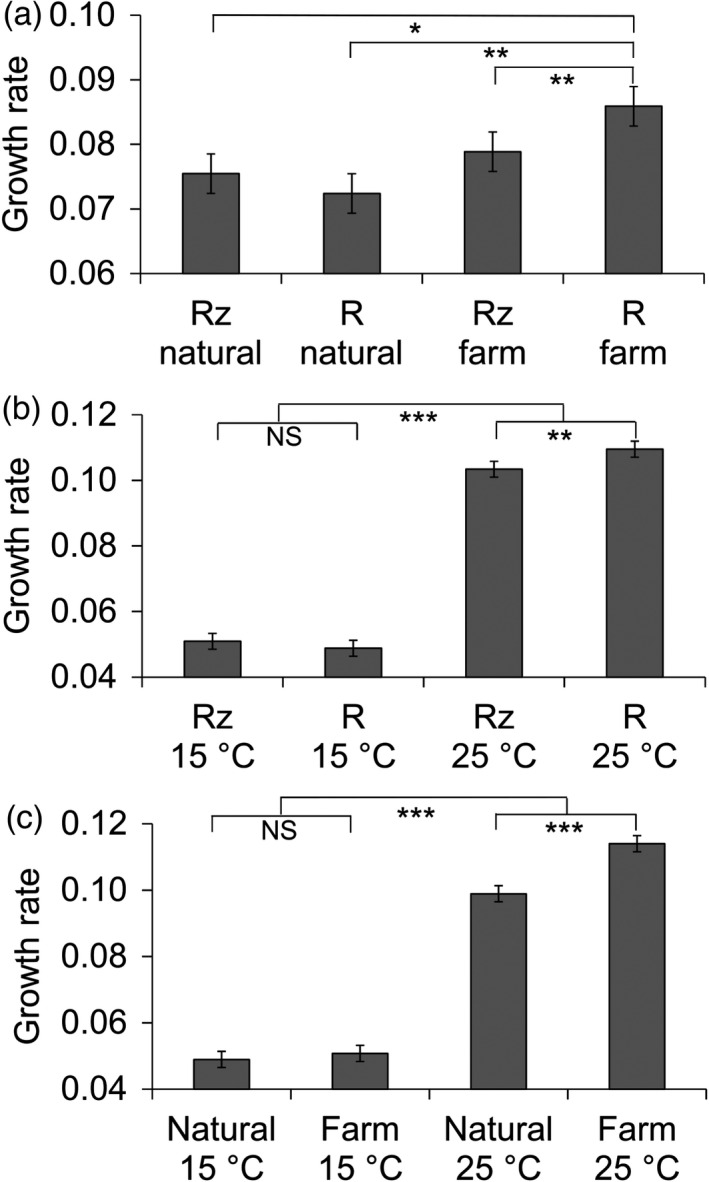
Effects of morphotype (Rz, rhizoid, R, rough) and origin (Natural, natural waters, Farm, fish farm) (a), morphotype and temperature (b), and origin of isolation and temperature (c) on maximal growth rate h^−1^ of *Flavobacterium columnare* (estimated marginal means ± *SE*). The growth rate was measured as a change in OD at 420–580 nm. Significant differences between treatments are denoted with *^,^ **^,^ ***; *p* < 0.05, *p* < 0.01, *p* < 0.001, respectively

The maximal bacterial growth rate was found to be affected by several factors (Table 2, Table S1). The growth rate was affected the most by the temperature, with the higher temperature supporting higher growth rates (Table 2). Growth rate also increased with resource concentration (0.05×, 0.1×, 0.5×, 1×, and 2× growth medium), although there were no statistical differences between the two lowest resource concentrations (*p* < 0.3 in pairwise tests). The rest of the pairwise tests indicated differences between resource concentrations (*p* < 0.001). The origin of isolate or morphotype affected growth rate only in interaction with other factors. Within fish farm isolates, rough morphotype (R) replicated faster than the rhizoid (Rz) (Figure 1a, *F*_1,620_ = 10.048, *p* = 0.002), whereas this was not evident within isolates from natural water (*F*_1,620_ = 1.902, *p* = 0.168), supporting our hypothesis of higher phenotypic variation in the fish farm isolates. Moreover, the growth rate of fish farm isolates was higher than the growth rate of isolates from natural waters only in the R morphotype (*F*_1,620_ = 9.772, *p* = 0.010), but not in the Rz morphotype (*F*_1,620_ = 0,620, *p* = 0.448). Growth advantage of R morphotype was found only at high temperature (*F*_1,772_ = 7.551, *p* = 0.006, Figure 1b), whereas at low temperature, there were no differences between the rough and rhizoid morphotype (*F*_1,772_ = 0.916, *p* = 0.339).

**TABLE 2:** Results of mixed model analyses on determinants of growth rate and yield in *Flavobacterium columnare* isolates

	Growth rate				Yield			
	*df*1	*df*2	*F*	*p*	*df*1	*df*2	*F*	*p*
Temperature	1	772	1298.417	<0.001	1	775	709.919	<0.001
Resource concentration	4	772	168.453	<0.001	4	775	1527.139	<0.001
Morphotype	1	772	4.411	0.069	1	775	6.621	0.010
Origin of isolate	1	8	1.603	0.206	1	8	0.017	0.899
Temperature × Resource	4	772	62.830	<0.001	4	775	74.572	<0.001
Temperature × Morphotype	1	772	6.863	0.009	–	–	–	–
Temperature × Origin of isolate	1	772	17.886	<0.001	–	–	–	–
Resource × Morphotype	–	–	–	–	4	775	6.984	<0.001
Resource × Origin of isolate	4	772	2.788	0.026	–	–	–	–
Morphotype × Origin of isolate	1	772	10.346	<0.001	–	–	–	–
Block	1	772	34.581	<0.001	1	775	2.958	0.086
Isolate’s ID	σ^2^ = 3.43 × 10^−5^, Wald Z = 1.695, *p* = 0.090				σ^2^ = 5.47 × 10^−3^, Wald Z = 1.931, *p* = 0.053			

Note

Clones were either isolated from fish farms or natural waters. From each clone, we measured two morphotypes (rough and rhizoid) in high and low temperatures (25 and 15°C) and five different resource concentrations (0.05×, 0.1×, 0.5×, 1×, and 2× Shieh medium). In statistical analyses, we also included effects of measurement block (identical measurements were done in two subsequent weeks), and maternal isolate's identity to control for nonindependence of observations arising from the shared genetic background. Excluded factor interactions are denoted with –.

**FIGURE 2:**
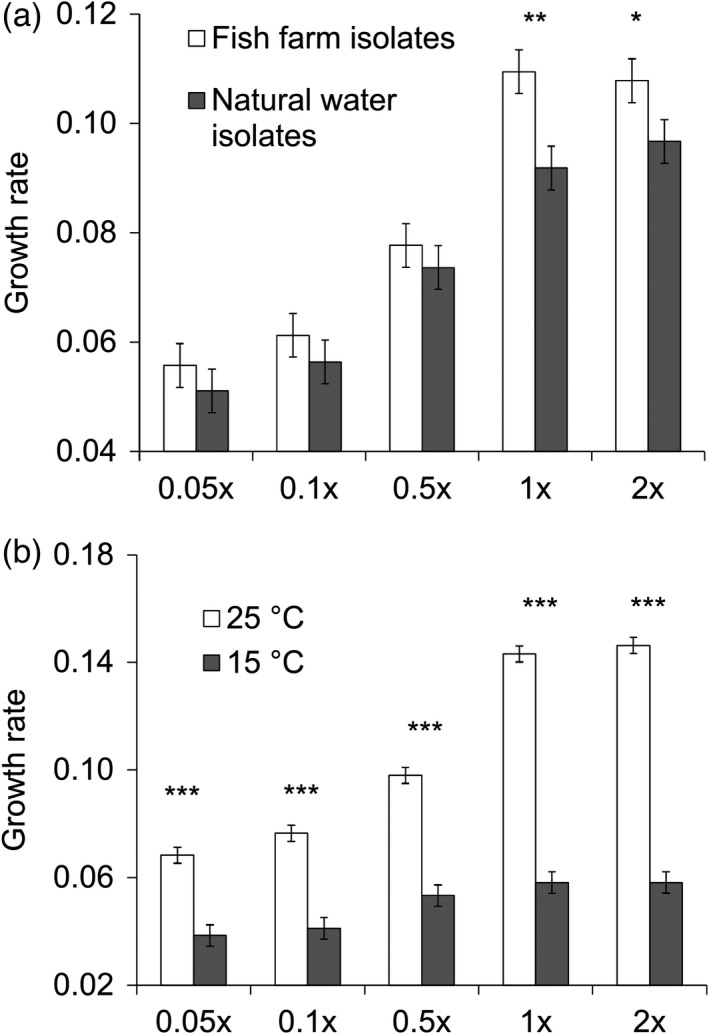
Effects of resource concentration (0.05×, 0.1×, 0.5×, 1×, and 2× Shieh medium) and origin of isolate (a) and temperature (b) on maximal growth rate h^−1^ of *Flavobacterium columnare* (estimated marginal means ± *SE*). The growth rate was measured as a change in OD at 420–580 nm. Significant differences between treatments are denoted with *^,^ **^,^ and ***; *p* < 0.05, *p* < 0.01, and *p* < 0.001 respectively

Farm isolates had higher growth rates than isolates from natural water in high temperature (Figure 1c, *F*_1,10.620_ = 12.209, *p* = 0.005), but not in low temperature (*F*_1,10.620_ = 0.176, *p* = 0.684). Farm isolates had significantly higher growth rates than isolates from natural waters in the two highest resource concentrations (Figure 2a, 0.05× medium: *F*_1,20.637_ = 0.826, *p* = 0.374; 0.1×: *F*_1,20.637_ = 0.906, *p* = 0.352; 0.5×: *F*_1,20.637_ = 0.630, *p* = 0.436; 1×: *F*_1,20.637_ = 11.882, *p* = 0.002, 2×: *F*_1,20.637_ = 4.740, *p* = 0.041).

Differences in growth rates between resource concentrations were larger in high temperature (Figure 2b): In high temperature, all pairwise tests between different resource concentrations were clearly significant (*p* < 0.006) except 0.05× vs. 0.1× (*p* = 0.203) and 1× vs. 2× (*p* = 0.999). In low temperature, differences between 0.05× and 0.1× (*p* = 0.999), 0.5× vs. 1× (*p* = 0.999), 0.5× vs. 2× (*p* = 0.999) were not found, whereas other pairwise comparisons yielded significant differences.

### 3.2 Yield

We found that biomass yield was best explained by a model with significant main effects of temperature, colony morphology and medium, and interactions between medium and temperature, and medium and colony morphology (Table 2, Table S1). Higher temperature led to higher biomass yield than lower temperature (yield at 15°C = 0.492, *SE* = 0.024; 25°C = 0.728, *SE* = 0.024, Table 2). In addition, Rz colony morphology (yield = 0.621, *SE* = 0.024) had higher biomass yield than R colony morphology (yield = 0.599, *SE* = 0.024, Table 2). The biomass yield was higher the richer the medium (*p* < 0.001 for all pairwise tests between different resource concentrations).

**FIGURE 3:**
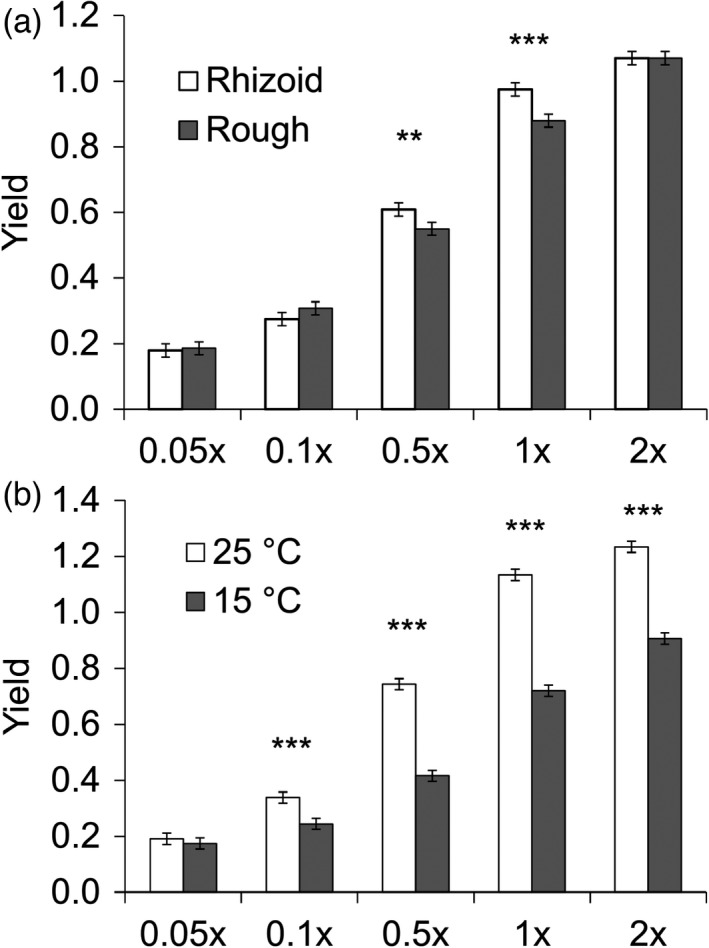
Effects of resource concentration (0.05×, 0.1×, 0.5×, 1× and 2× Shieh medium) and morphotype (a), and resource concentration and temperature (b) on maximal biomass yield of *Flavobacterium columnare* (estimated marginal means ± *SE*). The yield was measured as maximal OD at 420–580 nm. Significant differences between treatments are denoted with *^,^ **^,^ ***; *p* < 0.05, *p* < 0.01, *p* < 0.001 respectively

Rz morphotypes produced the highest biomass yield in intermediate resource concentrations (0.5×: *F*_1,775_ = 8.795, *p* = 0.003; 1×: *F*_1,775_ = 22.882, *p* < 0.001, Figure 3a). In the smallest and in the largest concentrations, colony types did not differ in their biomass yield (0.05×: *F*_1,775_ = 0.105, *p* = 0.746, 0.1×: *F*_1,775_ = 2.773, *p* = 0.096, 2×: *F*_1,775_ = 0.001, *p* = 0.995). Increase in resource concentration increased biomass yield, and the increase was larger in high temperatures (Figure 3b, Table 2. 0.05× resource: *F*_1,775_ = 0.688, *p* = 0.407, 0.1×: *F*_1,775_ = 22.458, *p* < 0.001, 0.5×: *F*_1,775_ = 273.742, *p* < 0.001, 1×: *F*_1,775_ = 437.327, *p* < 0.001, 2×: *F*_1,775_ = 273.993, *p* < 0.001).

### 3.3 Morphotype stability

**FIGURE 4:**
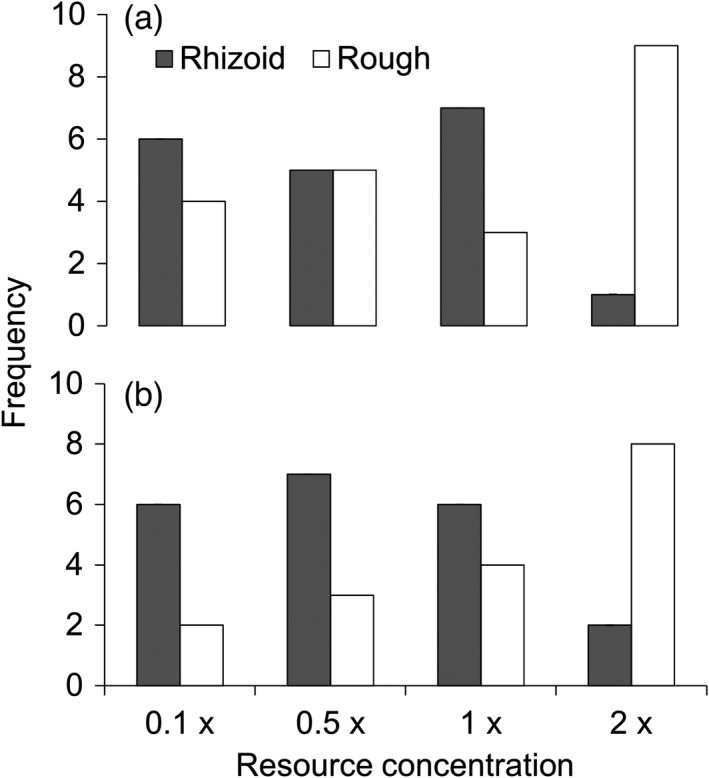
Stability of the rhizoid and rough colony morphotype on agar plates containing different concentrations of Shieh growth medium (0.1×, 0.5×, 1×, and 2×) after plating an equal number (five) of both colony types for isolates from natural waters (a) and fish farm isolates (b). Some of the rough morphotypes changed into rhizoid type in the three lowest resource concentrations, while Rz colonies changed into R type in the highest resource concentration. Note that one fish farm isolate did not grow on 0.1× Shieh concentration

When plated on agar plates containing the nutrient concentrations used in the growth experiment (0.1×, 0.5×, 1×, and 2× Shieh medium), some of the original rough colony morphotypes grew in a rhizoid form in concentrations from 0.1× to 1×. At the highest resource concentration (2×), the colony spreading decreased. The rhizoid colonies contracted and thickened, but some retained rhizoid edges (Figure S2). This change was more pronounced for isolates from natural waters (Fisher's exact test, *p* = 0.043) than for the fish farm isolates (*p* = 0.090; Figure 4). Plating on 0.05 × Shieh agar did not result in visible colonies for most bacterial cultures; hence, this treatment was omitted from statistical analysis.

### 3.4 Virulence test

**FIGURE 5:**
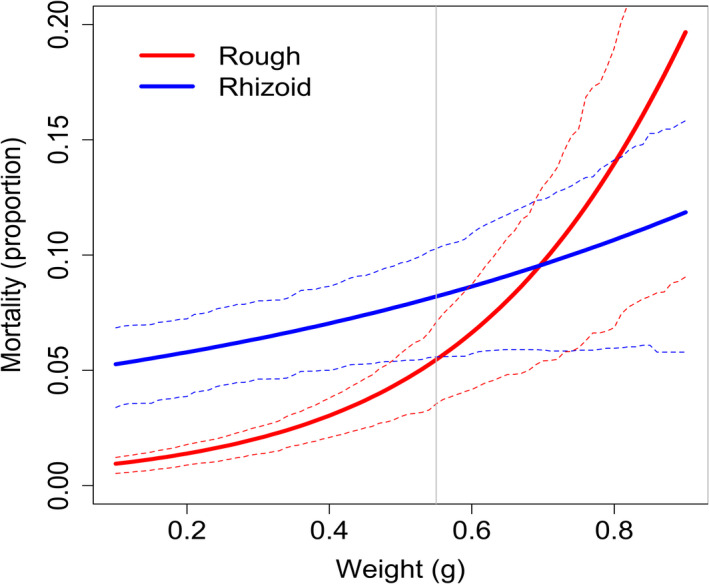
The effect of colony morphology of *Flavobacterium columnare* (blue: rhizoid, red: rough) and weight of the fish on fish mortality. Thin dashed lines indicate 80% credible intervals. The vertical line denotes critical weight (0.55 g) where, and below which, the mortality caused by rhizoid and rough colonies differ statistically significantly. Critical weight was approximated with the Johnson‐Neyman procedure (see Materials and Methods) implemented on predicted mortalities based on fish size, calculated using posterior distribution of estimates

In contrast to our hypothesis, isolate origin did not affect virulence. The best model explaining fish morbidity in the challenge experiment included the effects of colony morphology, fish weight, and their interaction (Table 3, Table S2). The virulence induced by the rhizoid morphotype was higher than virulence induced by the rough morphotype and increased with fish weight. For rhizoid colony type, the morbidity risk increased steadily with fish weight, while the morbidity risk induced by the rough colony morphology remained lower than that induced by the rhizoid type until a sharp rise at the largest fish weights (Table 3, Figure 5). Based on the modified Johnson‐Neyman procedure (see Materials and Methods), the mortality risk did not differ between morphotypes for fish weighing over 0.55 g. The isolates from gills of diseased fish were always rhizoid, even when the fish were challenged with the rough morphotype. All control fish survived until the end of the experiment. No *F. columnare* could be isolated at the end of the experiment from those fish who did not exhibit disease symptoms or from control fish. Rough morphotypes of the isolates B392 and B067 did not cause disease in exposed fish.

**TABLE 3:** The effect of colony morphology (type) and fish weight on the morbidity risk of the host fish in the virulence experiment

Source	Estimate	*SE*	*df*	χ^2^	*p*
Intercept	−3.05	0.3			
Type (rough)	−2.1	0.41	1	37.67	<0.001
Weight	1.08	0.62	1	20.99	<0.001
Type × weight	3.04	1.02	1	8.83	0.003

## 4 DISCUSSION

Human‐induced environmental change that favors different genotypic and/or phenotypic properties than the natural environment can have strong effects on organismal evolution (Baltazar‐Soares et al., 2021), including parasites and pathogens (Wolinska & King, 2009). Environmental changes particularly impact environmentally growing or transmitted pathogens because changes affect them directly and not only via effects on the hosts (Ashrafi et al., 2018; Brown et al., 2012). Phenotypic plasticity, the ability of a genotype to produce different phenotypes, has been considered important for adaptation to alternating environments for all kingdoms of life (DeWitt & Scheiner, 2004; Kristensen et al., 2020; Nussey et al., 2007; Scheiner, 1993). The phenotypic variation could be expected to be especially beneficial for environmentally growing opportunistic microbial pathogens for coping with selection pressures in two different environments, within and outside the hosts (Brown et al., 2012; Ketola et al., 2016). Here, we show increased phenotypic variation in an opportunistic bacterial pathogen in intensive fish farming, an environment that has been heavily modified by human activities compared to the natural environment. Contrary to our expectations, we did not find increased phenotypic variation in virulence in isolates from fish farms. Rather, high phenotypic variation in growth of the bacterial population together with high availability of outside‐host resources at high temperatures and simultaneous high availability of hosts could facilitate disease outbreaks at farms. The favorable environmental conditions for growth at fish farms along with antimicrobial treatments could therefore select high phenotypic variation in bacterial population and hence affect their evolution. Our results highlight the multifaceted effects of human‐induced environmental alterations in shaping epidemiology and evolution in microbial pathogens.

In clonally replicating bacteria, the heterogeneity of the phenotype can be achieved by inducible responses to environmental cues and by stochastic switching between alternative phenotypes produced by the same genotype (Ackermann, 2015; Balaban et al., 2004). In *F. columnare*, a plastic response to environmental change related to, for example, outside‐host resources or presence of fish hosts, is likely regulated by gene expression (Declercq et al., 2016, 2019; Laanto et al., 2012; Penttinen et al., 2018). Nutrient concentration has been found to affect expression of putative virulence genes and genes associated with gliding motility differently for the rhizoid and rough morphotypes of *F. columnare* (Penttinen et al., 2016, 2018). Loss of spreading and gliding motility in *F. columnare* as a response to adding cortisol, the stress hormone excreted by teleost fish hosts, has been suggested to be connected to biofilm‐forming ability on fish gills and hence infectivity (Declercq et al., 2016, 2019). We found that individual isolates varied in morphotype stability when plated on agar at different resource concentrations. Our findings support the hypothesis that morphotype change is inducible and related to nutrient searching. Variation in morphotype stability, altering resource and host availability at fish farms and environment are expected to affect the evolution of phenotypic change in *F. columnare*.

According to our results, the isolates originating from fish farms responded to higher temperature with increased growth as compared to isolates from natural waters. Temperature is one of the key factors affecting the occurrence of *F. columnare* epidemics at fish farms (Ashrafi et al., 2018; Pulkkinen et al., 2010). However, the difference in growth between fish farm and natural isolates is most likely not driven by a temperature difference between these two environments, because the farms take their water from natural water systems (Karvonen et al., 2010). Rather, our results point to a response of fish farm isolates to growth conditions driven by the interaction between temperature and other environmental factors, such as resource availability or medical treatments. Indeed, fish farm isolates responded to increasing resources more intensively than the natural isolates. Results pointing to this direction were found also in a previous study, which showed that *F. columnare* isolates in outlet water of a fish farm responded more to differences in resource concentration than isolates from inlet water (Sundberg et al., 2016). These results show the importance of considering interactions or correlations between the environmental variables when studying the effects of human‐induced environmental change in causing phenotypic change in microbial pathogens.

Increased nutrient supply in the outside‐host environment can directly affect the growth of opportunistic environmental microbes (Brown et al., 2012; Kinnula et al., 2017; Penttinen et al., 2016; van Elsas et al., 2011). At fish farms, the outside‐host environment can have high concentrations of fish feed and feces with protein‐rich substances 2–3 orders of magnitude higher than in the Shieh medium used in our growth experiments (Naylor et al., 1999). The accumulation of uneaten fish feed and excreta in the water is particularly evident in increased temperatures when the feeding activity of the fish is impaired (Ellis et al., 2002; Wedemeyer, 1996). The increased phenotypic variation in growth at farms was due to better growth of the less virulent rough morphotype of the fish farm isolates, suggesting that they are adapted to exploit the higher resource concentrations in water at farms in combination with increased temperatures. The resource‐rich environment at fish farms could facilitate increased phenotypic variation in growth by relaxing the costs related to maintaining multiple phenotypes (Friman et al., 2008; van Noordwijk & de Jong, 1986) and hence increase the adaptive value of phenotypic variation at farms (DeWitt et al., 1998; Koch & Guillaume, 2020).

The epidemiological conditions in intensive farming that select for fast growth and high transmission between hosts (Brown et al., 2012; Ebert, 1998; Frank, 1996) could be expected to select for both higher virulence and higher phenotypic variation (in virulence) between the morphotypes in farms. We did not find difference in virulence based on the origin of the isolates. Interestingly, our results suggest that the less virulent rough morphotype can revert into rhizoid type expressing high virulence when in contact with the fish. In the virulence experiment, rough morphotypes of several isolates induced host death, and all fish exposed to bacteria from rough morphotypes exhibited only rhizoid morphotypes in bacterial isolates from gills of diseased fish. The virulence induced by the bacterial population of the rough morphotype increased sharply with the weight of fish. At farms, growth on the organic waste from fish feed and feces that are nutritionally close to fish tissue could pre‐adapt bacteria for infecting fish (Ketola et al., 2016; Pulkkinen et al., 2018). A similar effect can be caused by mucins, the glycoprotein components of the mucosa on fish skin (Almeida et al., 2019). Rough type could be responding to the higher nutrient levels or mucins released by the larger fish hosts in the water or to a larger surface area for bacterial attachment and growth provided by the larger fish. The zebrafish model system used here has been shown to give a qualitatively similar response to bacterial doses and strains as the natural host rainbow trout (*Oncorhynchus mykiss*) (Kinnula et al., 2015, 2017), and therefore, we are confident that the results apply also to natural host settings. These findings suggest that conditions in farms do not directly select for phenotypic variation in virulence, but phenotypic variation in growth and morphotype reversibility in combination with environmental conditions favoring host invasion could select for higher phenotypic variation at fish farms.

In addition to maximal growth rate, bacterial performance and survival are affected also by yield, the amount of biomass that bacteria can accumulate with given resource quantity (Novak et al., 2006). Growth rate and yield are opposing metabolic strategies that are thought to be a trade‐off in microbes (Novak et al., 2006) and hence increased growth rate could indicate selection for the lower yield. For *F. columnare*, the high growth rate in the outside‐host environment is connected to high virulence in fish (Pulkkinen et al., 2010), and thus, the ability for fast utilization of outside‐host resources at fish farms could facilitate epidemics. On the other hand, high yield could be beneficial for environmental persistence outside fish hosts, both in natural environment with scarcity of fish hosts but also at fish farms during antibiotic treatments. The interaction between temperature and resource level for yield in our experiments suggests that given sufficient resources, *F. columnare* could continue to build biomass even in a temperature below its optimum, while the growth rate was strongly restricted by temperature regardless of resource level. Our results suggest that human‐induced environmental nutrient enrichment could sustain the environmentally growing opportunistic pathogens in the outside‐host environment also in suboptimal temperatures and increase the risk of epidemics when temperature increases.

In conclusion, our study suggests that intensive farming could act as an environment where the potential costs related to expression and maintenance of phenotypic variation are relaxed due to the availability of high resource concentration in the outside‐host environment (Friman et al., 2008; van Noordwijk & de Jong, 1986). In combination with this, the simultaneous high availability of hosts facilitates host invasion. Fish farms could therefore select for higher phenotypic variation and drive evolution in *F. columnare*. Our results contribute to the growing evidence of the role of intensive farming in inducing changes in the epidemiology of parasites and pathogens (Atkins et al., 2013; Mennerat et al., 2010, 2017; Pulkkinen et al., 2010; Sundberg et al., 2016) and of the potential of human‐induced environmental alterations in driving pathogen evolution. In addition, our results pinpoint the importance of considering determinants of not only the mean of the traits, but the variation in them, in depicting the selective role of environments.

## CONFLICT OF INTEREST

The authors do not declare competing interests.

## Supporting information

Fig S1‐S2

Table S1‐S2

## Data Availability

Data for this study are available at the Dryad Digital Repository https://doi.org/10.5061/dryad.b8gtht7cr.
