# Supplementary material for: Rich resource environment of fish farms facilitates phenotypic variation and virulence in an opportunistic fish pathogen
Source: Evol Appl. 2022 Feb 25;15(3):417–28. doi: 10.1111/eva.13355 (PMC8965373; doi:10.1111/eva.13355)
Supplement: Supplementary file 1 — Fig S1‐S2 [file EVA-15-417-s001.pdf]

Supplementary material for:

Rich resource environment of fish farms facilitates phenotypic variation and virulence in an opportunistic fish pathogen

Katja Pulkkinen, Tarmo Ketola, Jouni Laakso, Johanna Mappes & Lotta-Riina Sundberg

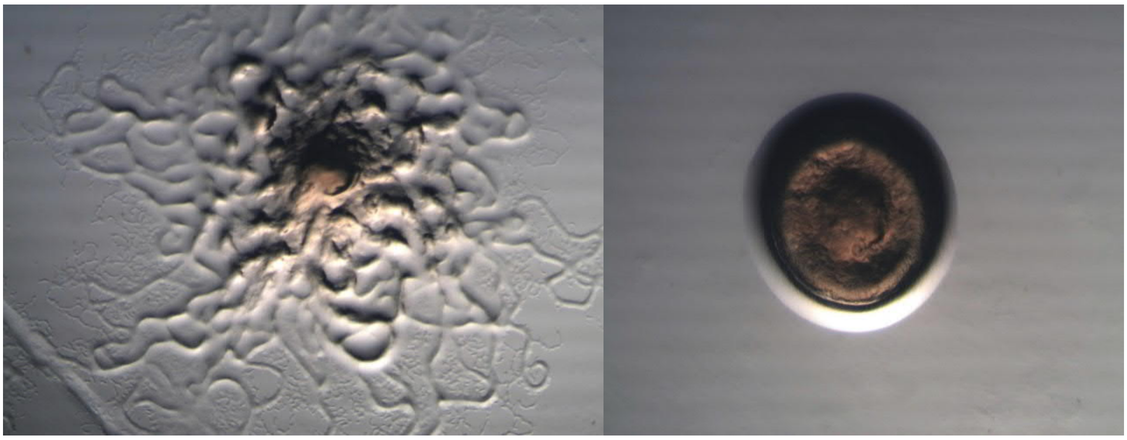

Figure S1. A rhizoid (left) and a rough (right) morphotype of *Flavobacterium columnare* cultured on Shieh-agar plates.

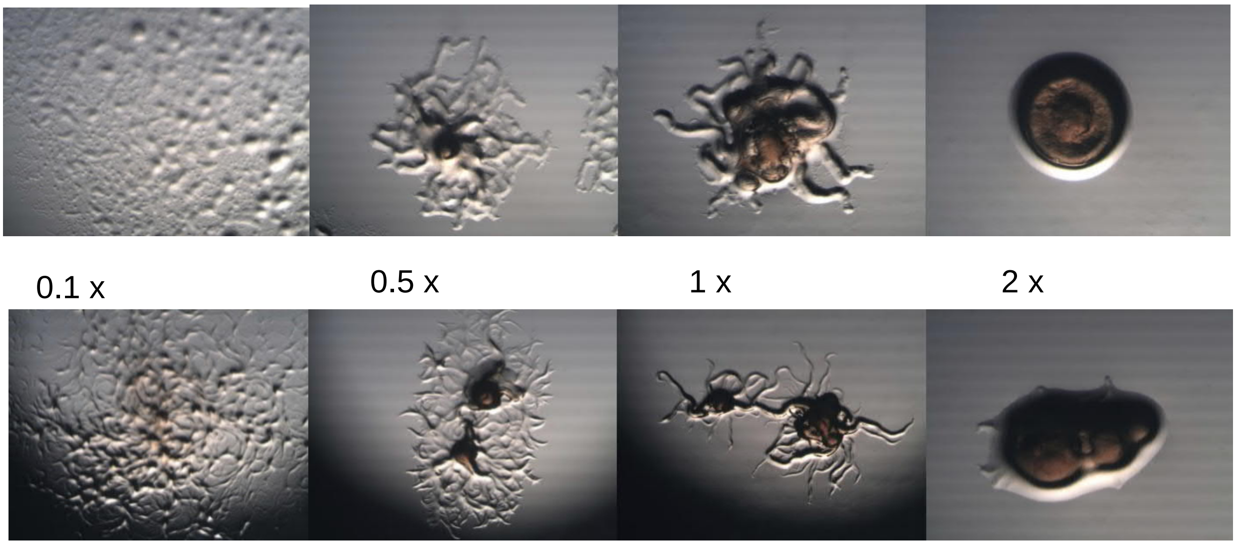

Figure S2. Examples of colony morphologies of *Flavobacterium columnare* plated on agar plates containing different concentrations of Shieh medium (0.1 x, 0.5 x, 1 x and 2x Shieh). Upper panel isolate E, lower panel isolate B067.
