# Supplementary material for: Rich resource environment of fish farms facilitates phenotypic variation and virulence in an opportunistic fish pathogen
Source: Evol Appl. 2022 Feb 25;15(3):417–28. doi: 10.1111/eva.13355 (PMC8965373; doi:10.1111/eva.13355)
Supplement: Supplementary file 2 — Table S1‐S2 [file EVA-15-417-s002.docx]

Supplementary tables for Rich resource environment of fish farms facilitates phenotypic variation and virulence in an opportunistic fish pathogen

Katja Pulkkinen, Tarmo Ketola, Jouni Laakso, Johanna Mappes & Lotta-Riina Sundberg

**Table S1.** Model selection for models explaining growth rate and yield based on AIC-criteria of fitted models (the smaller the value the better the model). Model fitting was done with backward model reduction by excluding non-significant parameters one by one, starting from highest order interactions. Models included main effects of temperature (T), resource concentration (R), morphotype (M) and origin of isolate (O), and their interactions. All models contained also fixed effect of measurement block (measurement week) and random effect of isolate’s identity, to control for non-independency of observations. Best models are highlighted with bold.

| *Models explaining growth rate* | -2LogL | AIC |
| --- | --- | --- |
| Main effects, all two-, three- and four-way factor interactions |  |  |
| Main effects, all two- and three-way factor interactions | -3533.73 | -3506.81 |
| Main effects and all two-way interactions + T×R×O + T×M×O + R×M×O | -3538.65 | -3534.65 |
| Main effects and all two-way interactions + T×R×O + T×M×O | -3563.47 | -3559.47 |
| Main effects and all two-way interactions + T×M×O | -3587.69 | -3583.69 |
| Main effects and all two-way interactions | -3591.56 | -3587.56 |
| Main effects + T×R + T×M + T×O + R×O + M×O | **-3625.64** | **-3621.64** |
| *Models explaining yield* |  |  |
| Main effects, all two-, three- and four-way factor interactions |  |  |
| Main effects, all two- and three-way factor interactions | -863.97 | -859.97 |
| Main effects and all two-way interactions + T×R×M + T×M×O + R×M×O | -877.23 | -873.23 |
| Main effects and all two-way interactions + T×R×M + T×M×O | -899.92 | -885.92 |
| Main effects and all two-way interactions + T×M×O | -900.80 | -896.80 |
| Main effects and all two-way interactions | -901.91 | -897.91 |
| Main effects + T×R + T×M + T×O + R×O + R×M | -907.80 | -903.80 |
| Main effects + T×R + T×M + R×O + R×M | -912.85 | -908.85 |
| Main effects + T×R + R×O + R×M | -917.51 | -913.51 |
| Main effects + T×R + R×M | **-932.89** | **-928.89** |

**Table S2.** Model selection of the virulence experiment based on Akaike information criteria (AIC). The best fit model estimating morbidity of the host (zebra fish) within time is marked with bold. O: origin (nature, fish farm), T: type (rhizoid, rough), W: fish weight (g), S: strain identity, + indicates the main effects, : indicates interaction.

| Model | AIC | DF |
| --- | --- | --- |
| O+T+W+O:T+O:W+T:W+O:T:W+(1\|S) | 488.9 | 9 |
| O+T+W+O:T+O:W+T:W+(1\|S) | 487.41 | 8 |
| O+T+W+O:W+T:W+(1\|S) | 485.44 | 7 |
| O+T+W+O:T+T:W+(1\|S) | 485.41 | 7 |
| O+T+W+O:T+O:W+(1\|S) | 494.05 | 7 |
| O+T+W+T:W+(1\|S) | 483.44 | 6 |
| **T+W+T:W+(1\|S)** | **481.72** | **5** |
| T +W+ (1\|S) | 488.26 | 4 |

As ΔAIC was less than 2 between the best fit model and a model containing the effect of origin (O+T+W+T:W+(1|S)), we checked whether term “origin” should be considered as an explanatory variable. As the term was statistically not significant (Estimate 0.20, SE 0.37, Chi sq. 0.28, p = 0.593), we concluded that the model containing colony type, fish weight and their interaction to best explain fish mortality risk.
